# MIMO antenna array with the capability of dual polarization reconfiguration for 5G mm-wave communication

**DOI:** 10.1038/s41598-022-23163-3

**Published:** 2022-10-31

**Authors:** Farzana Arshad, Ashfaq Ahmad, Yasar Amin, M. Ali Babar Abbasi, Dong-you Choi

**Affiliations:** 1 Telecommunication Engineering Department, University of Engineering and Technology, Taxila, 47050 Pakistan; 2 Communications and Wave Propagation Laboratory, Department of Information and Communication Engineering, Chosun University, Gwangju, 61452 South Korea; 3 School of Electronic Engineering and Computer Science, Institute of Electronics, Communications and Information Technology, Queen’s University, Belfast, UK

**Keywords:** Electrical and electronic engineering, Electronic and spintronic devices

## Abstract

This communication presents a polarization reconfigurable antenna array (PRAA) with Multi-input Multi-output (MIMO) formation for 5th generation (5G) millimeter wave (mm-Wave) communications. At first a single corner curtailed diagonal slotted cylindrical patch is printed on Roger RT Duriod 5880 and the overall size of the single antenna is 12 × 12 × 0.787 mm^3^. The circular polarization (CP) is realized by adding the diagonal slot in the circular patch. The antenna design is extended into two elements antenna array which occupies 20 × 20 × 0.787 mm^3^ footprint. The collection is formed using a T-shaped power divider/combiner. Pin-diodes are integrated with the patches to switch the polarization state between LP (Linear polarization) and CP radiation. The edge-to-edge distance between antenna elements is 6 mm. The design covers the 25.2–29.4 GHz band, and the maximum peak gain is 11.5 dBi. Moreover, a two-port (2 × 2) MIMO design is formed to increase the channel capacity. To isolate the ports, a sin-like slot is engraved in the ground, defected ground structure (DGS) technique of mutual coupling reduction; it can easily be implemented and increases the design efficiency. The port isolation is well above 30 dB for the entire operating band. Moreover, the Mean Effective Gain (MEG), Diversity Gain (DG), and Envelope Correlation Coefficient (ECC) are investigated, which are key performance metrics of MIMO. A prototype of the realized MIMO antenna system is fabricated, and the simulated outcomes carried out by Computer Simulation Technology (CST) tools are validated by experimental findings.

## Introduction

5th generation wireless communication utilizes the mm-wave frequency bands to return the unprecedented data traffic growth. For the 5G new radio (NR), the 3GPP in release 15 had specified the four frequency bands in the frequency range 2 (FR2). The quality of a simultaneous massive number of mobile connections is ensured by reserving the wideband channels^1,2^. Signal strength can be increased utilizing the antenna array, but the array possesses the same channel capacity as a single antenna. Link reliability, spectral efficiency, capacity, and improved data rate can be achieved by deploying the MIMO antenna systems at the transmitting and receiving end, which offers various paths for data traffic. Circular polarization (CP) antennas are crucial to immune the multipath effects, interference, and fading^3–5^.

As mm-Wave frequency bands plague great path loss antenna arrays and multi-input and multi-output (MIMO) antenna systems are highly needed for 5G technology to combat sever path loss. Mainly linearly polarized (LP) antenna designs for the 5G (26/28 GHz) band are reported, which can propagate only in one direction. However, a circularly polarized (CP) antenna can transfer with the same signal strength in two orthogonal directions consequently transmitting and receiving antennas have freedom of orientation. Supplementary, CP antennas are immune to propagation losses, interferences, and multi-path distortions, which are significant limitations of mm-wave frequencies. A low-profile broadband SIW 3-dB coupler with the same amplitude and 90-phase gap^6^ feeds the SIW H-plane horn and Vivaldi antennas. The 3-dB measured axial ratio spans the 24.25–26.5 GHz band, with the 8.4 dB gain. A SIW-based CP tapered slot design is reported in^7^, which can operate from 80.1 to 108 GHz, having an axial ratio < 3 dB and a maximum gain of 7.9 dBi. A sequentially rotating phase (SRP) multi-layer substrate-integrated gap waveguide (SIGW)-fed 2 × 2 CP patch antenna array configuration while covering 3 dB AR bandwidth from 22.8 to 27.6 GHz is demonstrated with the highest gain of 11.53 dBi in^8^. In^9^, a wideband CP element with a SIW 90° directional coupler is built by integrating two orthogonal slots. This design AR is < 2 dB and the maximum gain is 10.5 dBi. A 4 × 4 slot antenna array with an antipodal curvedly tapered shape is demonstrated to produce the CP radiations^10^. The AR bandwidth is from 81 to 95 GHz, and the maximum gain is 18.5 dBic. In^11^, utilizing a low-temperature cofired ceramic (LTCC) technology, a CP patch antenna capable of covering a 3-dB AR of 26% and a maximum gain of 9.3 dBi is proposed.

A 3D printed polarizer is proposed to transform the polarization from linear to circular^12^. The 3D printed polarizer consists of multiple air and dielectric slabs to attain linear to circular polarization. It achieves 30% AR from 49 to 67 GHz with a maximum gain of 15dBic. To accomplish the broadband CP radiation, a polarizer is realized and placed in front of the horn antenna^13^. The design can cover a CP radiation bandwidth of 22–29.8 GHz with a maximum gain of 7.8dBi. In^14^, 4 × 4 cavity-backed slot antenna array quadruple ridge waveguide polarizers and SIW T-type power dividers are used to obtain the 3 dB axial ratio from 35.3 to 35.55 GHz with a maximum gain of 18.4 dBi. A novel design of polarizers is introduced for generating circular polarization (CP) in a Fabry-Pérot Cavity (FPC) antenna. For the first time, a 3D-printed polarizer was combined with an embedded partially reflective surface and spans the 54.5–66.7 GHz frequency band^15^.

The efficiency of antennas, such as impedance bandwidth, AR, gain, and linear to circular polarization, has recently been enhanced using metasurface (MS). A multilayer wideband CP antenna array (4 × 4) with an H-shaped SIW power divider is realized in^16^. The AR and impedance bandwidth are enhanced with MS. An AR bandwidth from 39.2 to 51.5 GHz and the highest gain of 18.2 dBic is accomplished. In^17^, a diagonal slotted antenna is sandwiched between grounds and metasurface, which contains 4 × 4 equal square rings. Consequently, the broad 3-dB AR frequency range (24.1–29.5 GHz) is accomplished with a maximum gain of 11 dBi.

CP is also achieved by different shapes of dielectric resonator antennas (DRA) with varying mechanisms of feeding^18–20^.

AR was increased using the multifold mechanism, but this mechanism requires an external power divider. As a result, systems structure become large and complex^21^. AR bandwidth is also increased in some CP DRA using hybrid cross slots^22^, cross slots^23^, and spiral slots^24^, which is the easiest and simple way to use with traditional DR.

CP reconfigurable antenna is proposed^25^. The structure of the antenna array of 4 elements is indicated for 5G with the ability to adjust its function between the two CP modes. The acquired AR bandwidth spans the 27.2–28.35 GHz frequency band. All the above-mentioned designs do not encompass the MIMO feature.

Few CP antenna designs with the MIMO features are reported in the literature for 5G MMW communications. The high gain and high isolation are provided by the 5G MIMIO Fabry–Perot CP antennas^26,27^ but encompass the immense antenna profiles, intricate designs, and the air gap between superstrate and radiator cases mechanical issues. Partial reflective surfaces are employed to enhance the gain and AR of a CP MIMO antenna^28^. However, the design realization and mass production of manifold coated substrates/FSS (frequency selective surfaces) for practical applications is difficult.

In^29^, the LP and CP radiations are presented by twelve ports MIMO antenna for the 5G base station. Based on the user requirement the antenna system transmits the LP and CP radiation. The single element achieves the 8 dBi peak gain and 3% (27.5–28.5 GHz) AR bandwidth. In^30^ polarization agility is acquired by exploiting the antenna's different orientations, leaving the antenna geometry to be altered. The reconfiguration feature is not incorporated in these MIMO designs.

It can therefore be inferred that none of the antennas itemized in the literature have the advantages of simultaneously providing broad bandwidth, high gain, polarization diversity, and MIMO features. Currently, it is needed to offer a CP MIMO antenna capable of being low profile and operating on the allotted 5G frequency range. Considering the above a single-layer, reconfigurable CP MIMO for 5G communication systems is proposed.

Reducing the electromagnetic coupling energy between MIMO elements is vital to enhance the MIMO antenna performance. While designing the MIMO antenna isolation is a crucial parameter to be considered. The researchers have introduced multiple isolation or decoupling techniques in the literature to reduce these effects. These techniques are an unavoidable part of MIMO antenna design and play a vital role enhancing the MIMO system's performance. There are multiple mutual coupling reduction techniques like Neutralization Lines, Defected Ground Structures, Decoupling networks, Met material, Dielectric Resonators, Complementary Split Ring Resonator (CSSR), Slot or Parasitic Elements and Frequency Switchable Antennas, etc.^31–33^. The metallic via-holes can be connected to the ground to isolate the individual radiating elements which improves the isolation. SIW properties can be utilized to realize the via-holes^34^. A fractal isolator can also be used to reduce the mutual coupling^35^. The fractal isolator can be etched between two antennas. Through the substrate media and the space above and below them, two patch antennas are electromagnetically coupled. Contrary to other traditional methods of mutual coupling suppression where a decoupling slab is placed between the radiating antennas, it is also possible to embed the linear slots close to the patch's edge^36^. However, DGS method is used in this communication which is simple and can easily be implemented.

This paper presents two ports MIMO with two cylindrical slotted shape antenna arrays. The antenna array work as a linearly polarized and circularly polarized antenna. Diagonal slots are inserted to achieve circular polarization. The Pin-diodes are incorporated within the antenna structure to switch the radiation state of the antenna array between linear polarization and circular polarization.

### Design configuration

**Figure 1:**
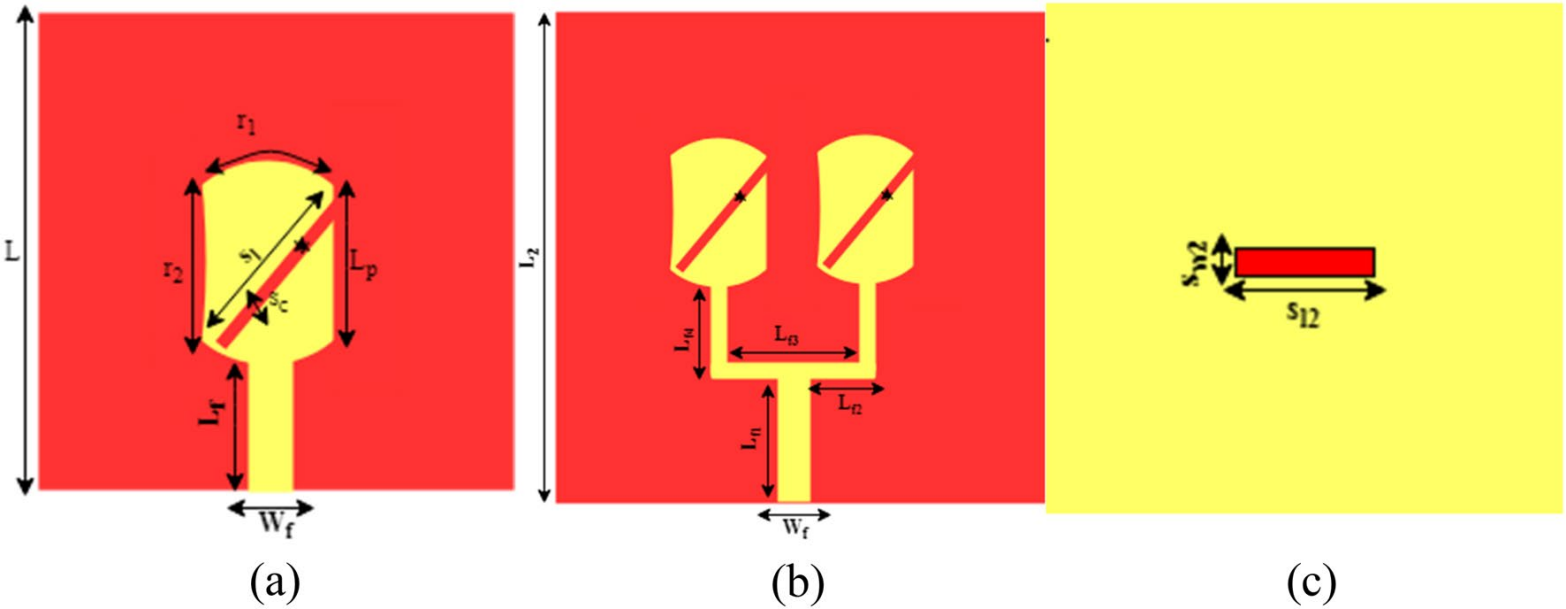
(**a**) Geometry of single element. (**b**) Antenna array front view. (**c**) Antenna array back view.

The geometry and structure of the PRAA are depicted in Fig. 1. The design is started from a circular patch and the radius of the patch is calculated using conventional equations of circular patch antenna^37^. A slanted slot with one open end is introduced in a circular patch. A cylindrical shape is obtained by truncating the side edges of a circular patch. Antenna is printed on substrate Rogers RT/Duroid 5880 (εr = 2.2, tanδ = 0.0009, h = 0.787 mm). Moreover, Copper is used as a ground to back the substrate. The parameters are tabulated in Table 1.

**Table 1:** Parameters of proposed design.

Parameters of single element							
L	W_f_	L_f_	r_1_	r_2_	s_l_	s_c_	L_p_
12	2.4	4	3	2	5.8	0.2	4

Parameters of array element							
L_2_	L_f1_	L_f2_	L_f3_	L_f4_			
20	4.5	2.5	6	3			

Parameters of MIMO							
W	s_w_	s_l_	T				
50	0.2	Sin(t)	− 10:10				

Then two elements antenna array is formed to increase the gain, as shown in Fig. 1b.

The overall design size is 20 × 20 × 0.787 mm^3^. A circular patch has linear polarization. A rectangular diagonal slot is inserted to acquire the CP radiation. The width of the slot is calculated using the following equation^38^.

$$ {\text{s}}_{{\text{c}}} = {\text{L}}_{{\text{p}}} /27.2 $$

However, the optimized length of the slot is achieved after running a few simulations. The corners of the patch are truncated which increases the gain of the patch antenna.

**Figure 2:**
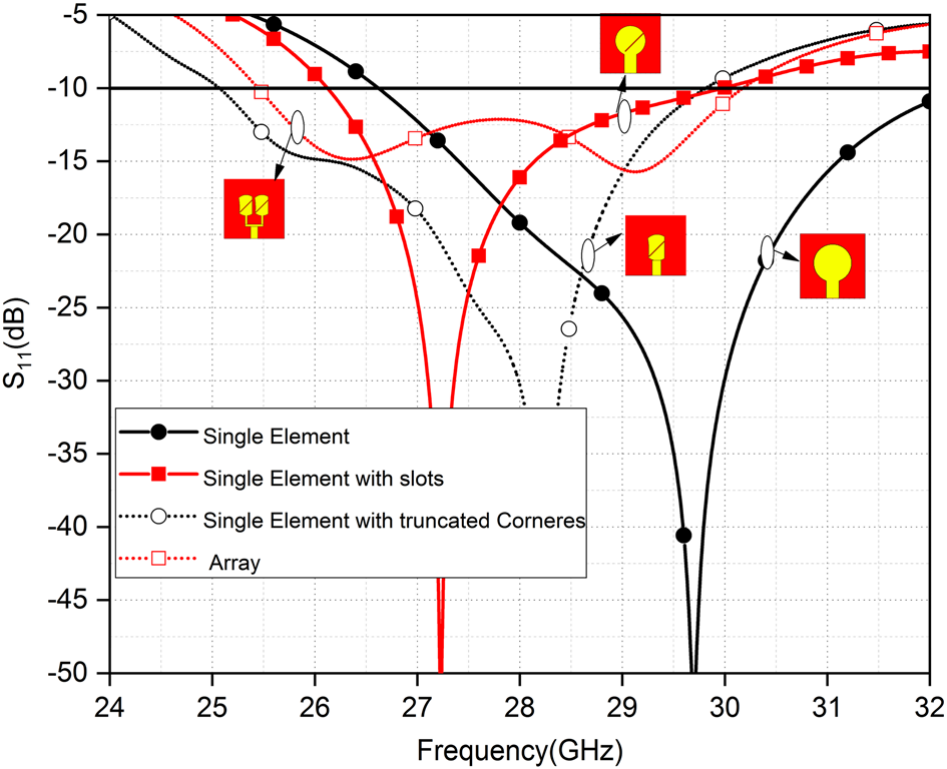
Reflection coefficient of design evaluation.

Figure 2 indicates the − 10 dB impedance bandwidth of design at different stages. A circular patch without a slot can operate from 26.7 to 32.2 GHz and an antenna with a slot has impedance bandwidth from 26.2 to 30 GHz. However, after truncation of sides of patch with slot the impedance from 25.2 to 29.7 GHz is achieved. The 25.5–30 GHz band is covered by the PRAA. The design evaluation has a different − 10 dB impedance bandwidth but covers 26.5–29.5 GHz which is the n257 band specified by 3GPP in release 15’ for 5G communications.

**Figure 3:**
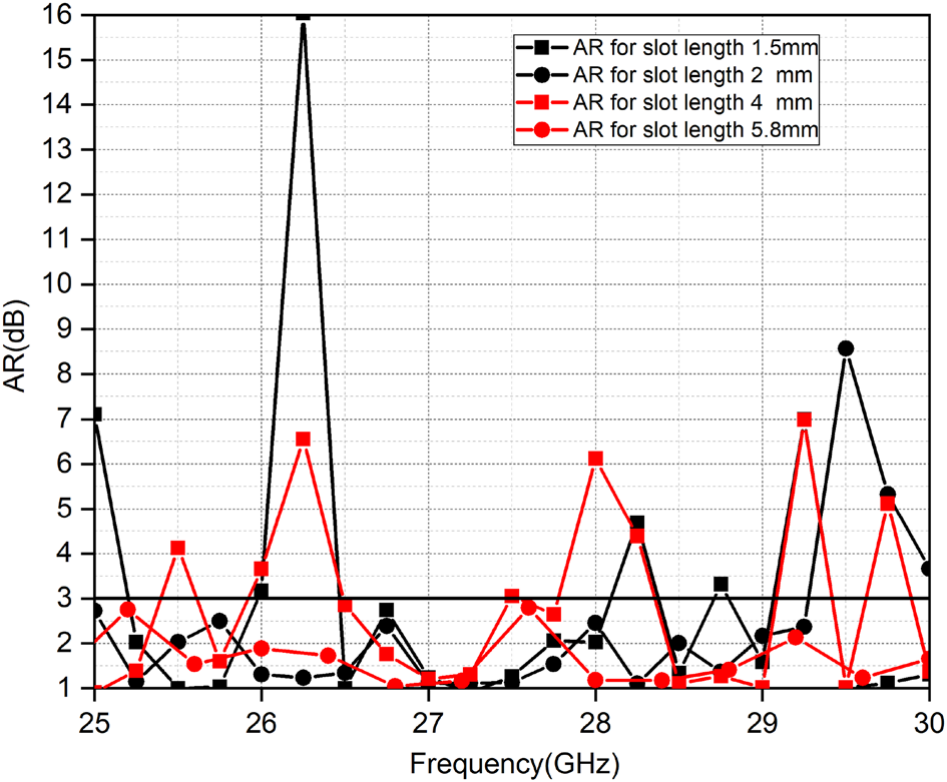
Parametric Analysis of the impact of slot length on AR.

The crucial parameter to measure the polarization is AR, which is usually below 3 dB for CP. Figure 3 show the parametric analysis of a slot length as due to insertion of this diagonal slot CP is achieved. The axial ratio varies with the slot length. The AR below 3 dB over the whole desired band is achieved for slot length of 2 mm.

**Figure 4:**
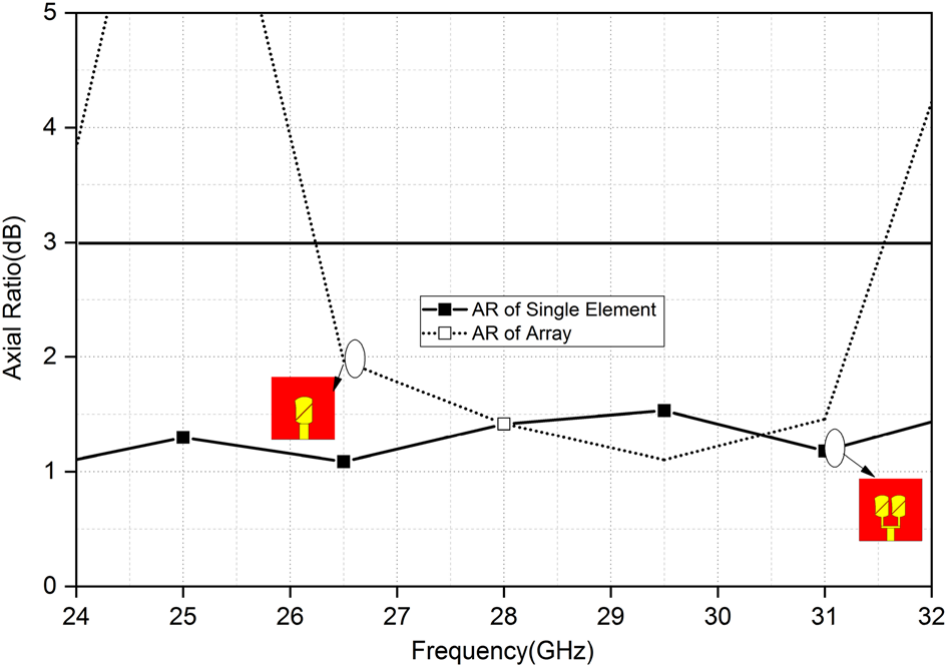
Axial ratio of single element and PRAA.

Figure 4 shows the AR of a single antenna and antenna array. The 3-dB axial ratio of the single antenna is from 26.6 to 31.5 GHz. The 3-dB AR bandwidth of the array is wide as compared to a single element. It is worth noting that the whole worldwide 5G mm-Wave frequency band (26.5–29.5 GHz) is cap by the operational CP bandwidth of the PRAA. Around 26.5 GHz the 1 dB minimum AR value is yielded by PRAA.

**Figure 5:**
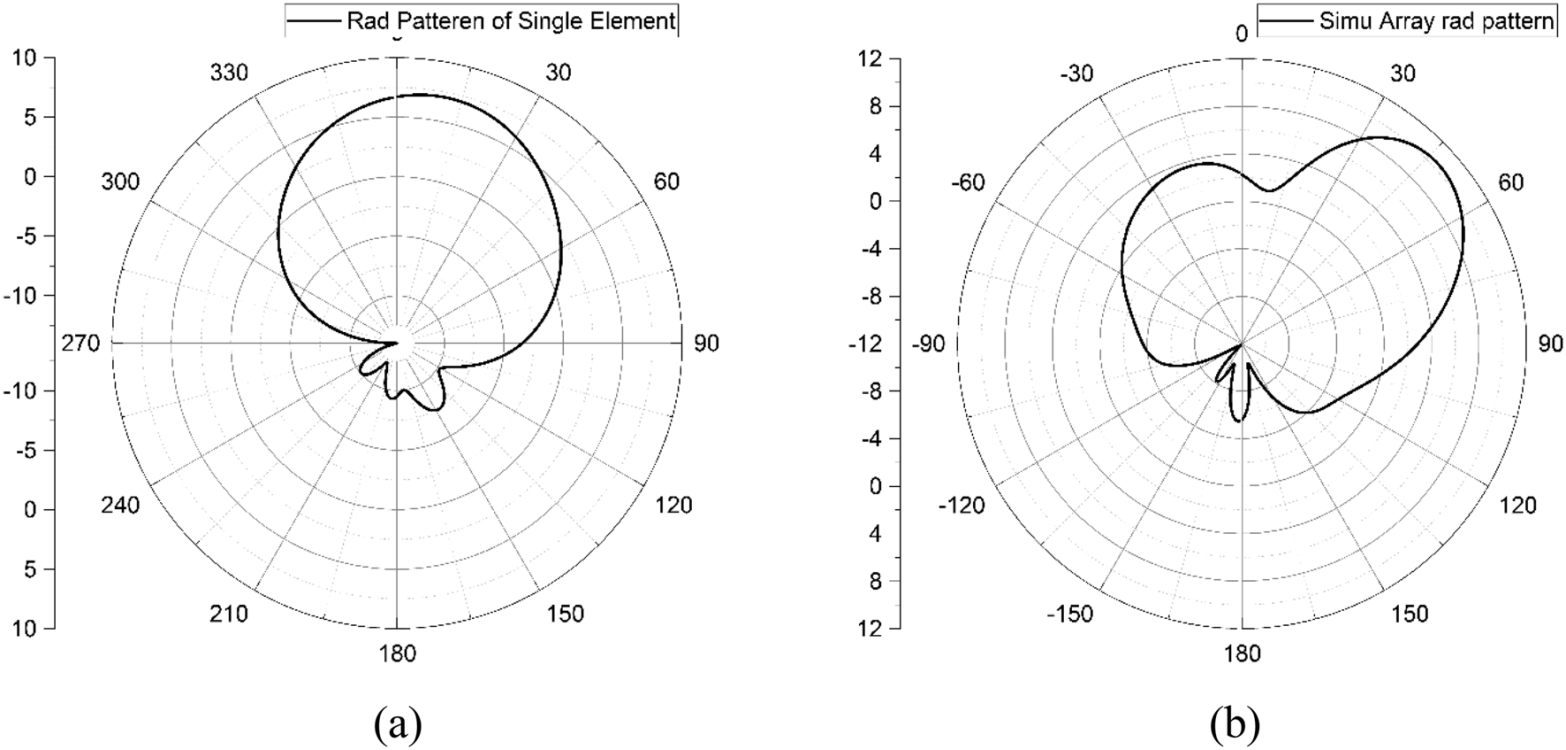
XZ plane (H-Plane) simulated radiation pattern of single element and antenna array.

The radiation pattern of design evaluation is shown in Fig. 5. The simulated radiation pattern of a single element is tilted towards 11°, and the maximum gain is 7.5 dBi. The simulated radiation pattern is tilted towards 50°, with a peak gain of 11.4 dBi.

**Figure 6:**
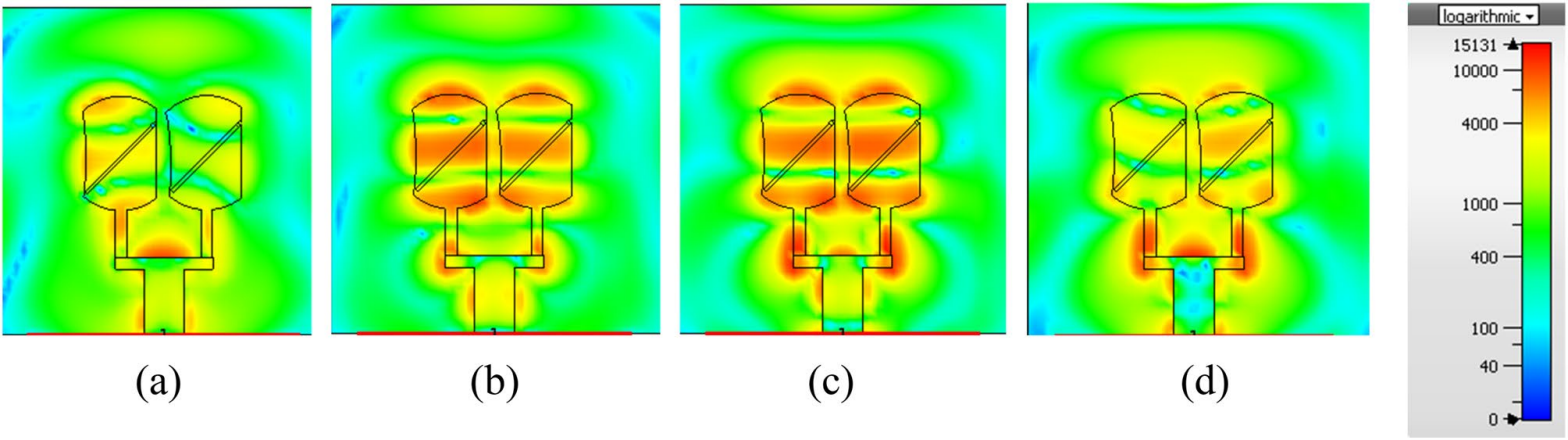
Variations of the E-field vs. various phases (**a**) 0°, (**b**) 90°, (**c**) 135°, (**d**) 180°.

**Figure 7:**
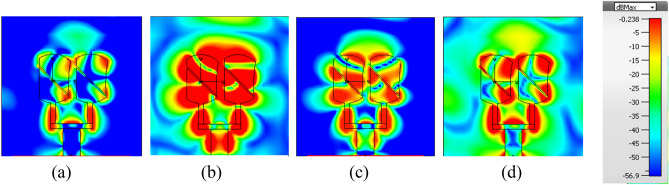
Variations of the E-field vs. various phases (**a**) 0°, (**b**) 90°, (**c**) 135°, (**d**) 180°.

Figure 6 shows the E-field directions observed from + z directions. The figure illustrates that the E field rotates in a counterclockwise direction which means that the antenna array is right-hand circularly polarized (RHCP). The direction of the slot is changed to observe the E-field direction. In the second case slot is left-oriented and has the same polarization. The same E-field rotation is observed as can be seen in Fig. 7.

**Figure 8:**
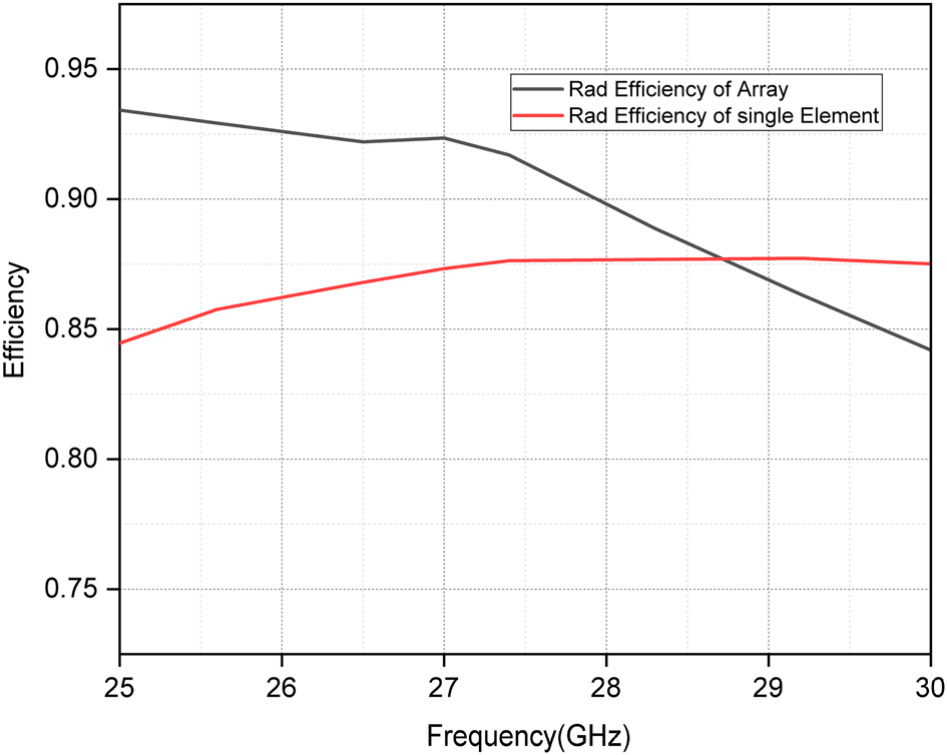
Radiation efficiency of single antenna and antenna array.

Figure 8 shows the radiation efficiency of a single antenna and antenna array. It can be observed that the radiation efficiency of the antenna array is greater than the efficiency of a single antenna below 28.8 GHz. The radiation efficiency of a single antenna increases with the frequency.

### MIMO configuration

**Figure 9:**
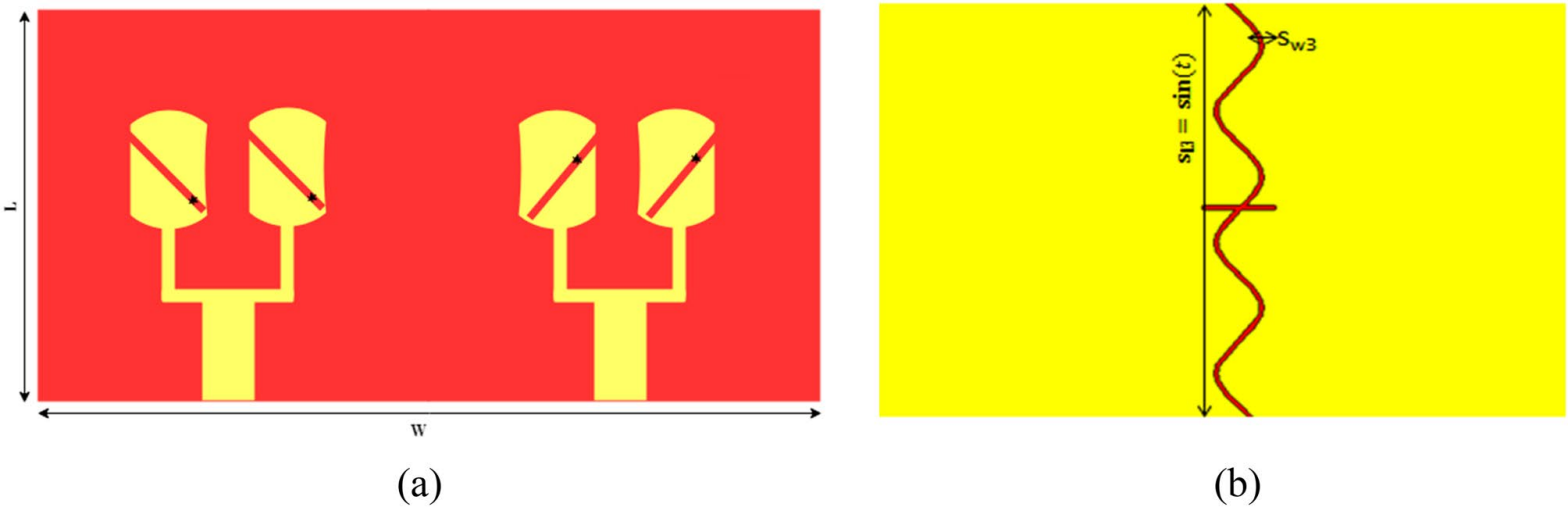
(**a**) Front view of MIMO. (**b**) Back view of MIMO with sin (t) slot.

To exploit the multipath propagation to enhance the data rate, capacity, and link reliability for the array, the design is further integrated into 2-port MIMIO to make it applicable for 5G MIMO applications. Figure 9a, b illustrates the MIMO configuration. The isolation between MIMO elements, return loss, envelops correlation coefficients, and diversity gain is investigated, which are performance parameters of MIMO. Elements isolation in the MIMO system is realized through the DGS method. Consequently, port isolation is enhanced with minimum effects on other performance parameters. A sin wave, like a vertically oriented slot, is introduced in the ground as depicted in Fig. 9b.

The Proposed MIMO has the capability of Polarization reconfiguration. The 4 PIN diodes are integrated for switching purposes. The fast switching speed and low weight are the features of PIN diodes. However, their integration potential is limited with antennas due to insertion losses and actuating voltages. When the PIN diode is forward-biased, it allows RF energy to flow, and reverse-biased blocks RF energy.

This is the basis for using the PIN diode in various of RF switch topologies. The electrical model of the PIN diode looks like an inductor in series with a resistor when forward biased. When reverse biased, it looks like an inductor in series with a paralleled capacitor and resistor. The specific values of the passive elements in these models depend upon the PIN diode model.

**Figure 10:**
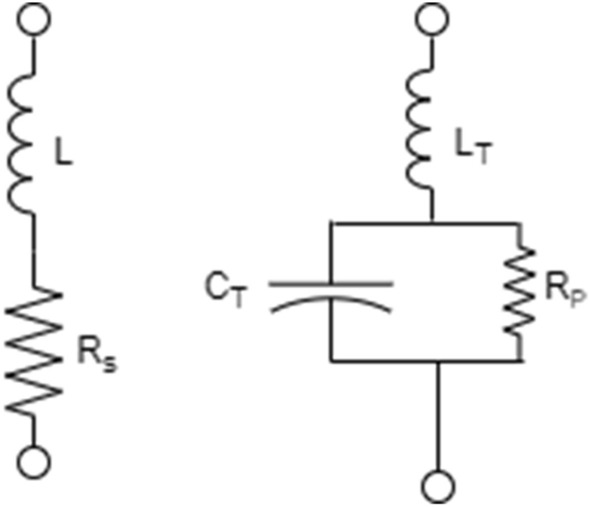
Circuit model of PIN diode in ON and OFF states.

Above Fig. 10 shows the forward and reversed biased mode of the PIN diode. L and Rs represent parasitic inductance and low resistance in reverse bias. CT represents the parasitic capacitance of the package and diode junction capacitance sum. In the reverse bias, Rp is high resistance.

The diode is only a core switching element. Without using other components, it’s not a complete and helpful switch. DC blocking capacitor and RF choke is a minimum requirement for a switch. The capacitor can block the DC bias current to reach the RF output and RF chock to provide a path for the DC bias current to return while blocking the RF signal. Four PIN diode MA4AGBL912 switches are used. These switches can work up to 40 GHz. The switches have been configured, as shown in Fig. 10.

**Figure 11:**
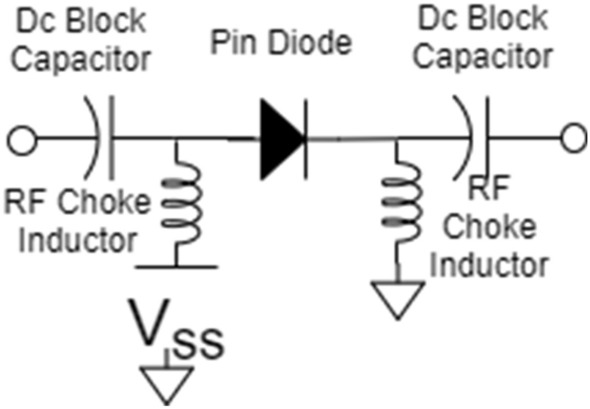
Biasing circuit.

Considering ON and OFF switch combinations, significant polarization changes are observed. Figure 11 shows the diode model^39^.

The resistive, capacitive, and inductive parameters of the switches have been adapted from the product datasheet. The Rs = 4.2 Ω, and Ls = 0.5 nH are series resistance and inductance corresponding to the ON state of the PIN diode. A series capacitance Ct, equal to 28 fF, and inductance is given by Ls = 0.5 nH corresponds the off state of the PIN diode.

## Results and discussions

**Figure 12:**
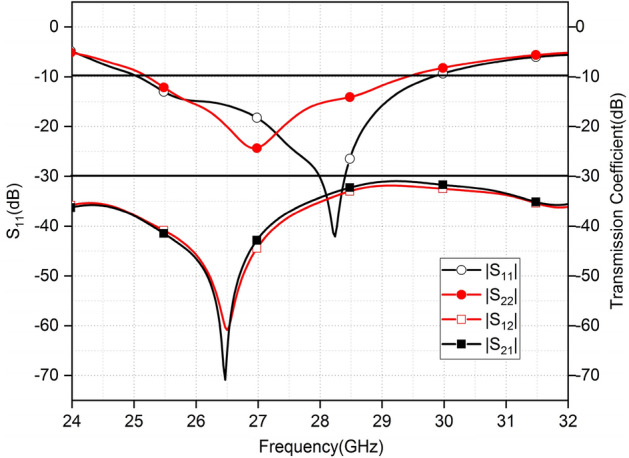
Reflection coefficient and transmission coefficient of MIMO.

The − 10 dB impedance bandwidth demonstrated by the MIMO antennas is 25.2–29.5 GHz. The |S11| and |S22| have different curve shapes and dips at different frequencies but both cover the global 5G mm-wave band. It can be noted from Fig. 12, the transmission coefficient indicating isolation between the MIMO elements is < 32 dB. As the antenna arrays are identical and symmetrical the simulated |S12| and |S21| are the same in the entire operating frequency range.

**Figure 13:**
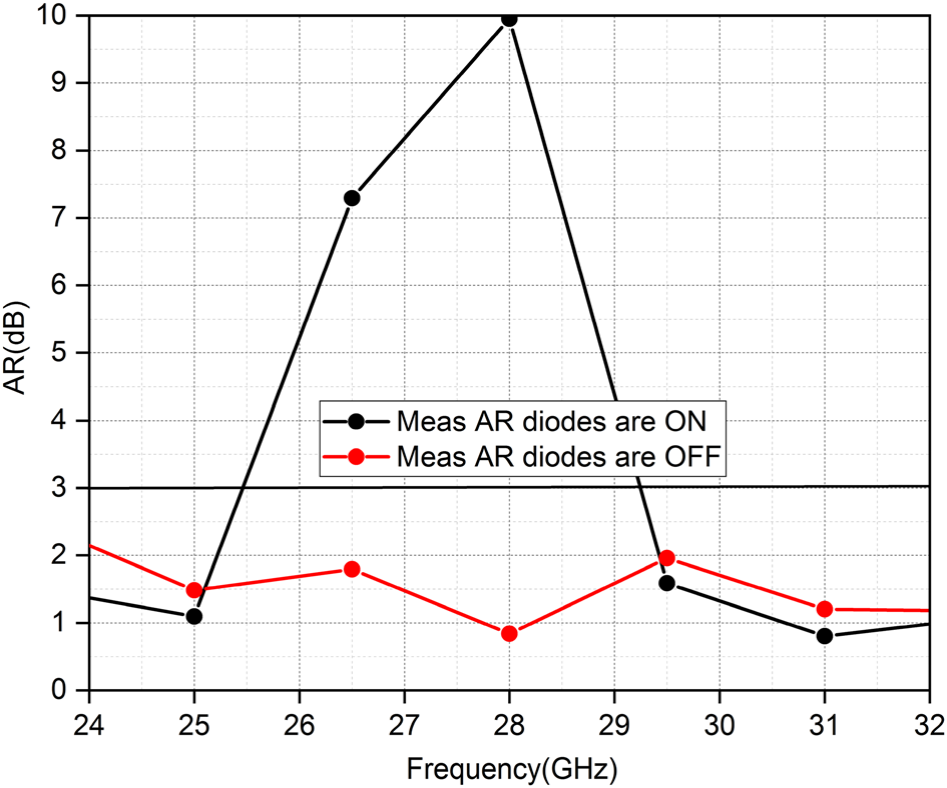
Simulated AR in both states of diodes.

If we change the length of the slot in the patch the axial ratio changes (AR) accordingly. Pin-diodes are integrated with each slot to utilize the effect of variation in slot length. The length of the slot varies with the ON and OFF states of diodes; consequently the orientation of electric fields changes which leads to two polarization states, i.e., circular polarization and linear polarization. When the diodes are ON the antennas radiate a linear polarization; when diodes are OFF MIMIO antennas generate CP radiations. Figure 13 shows that 3-dB AR bandwidth occupies the whole operating frequency band of the proposed antenna.

**Figure 14:**
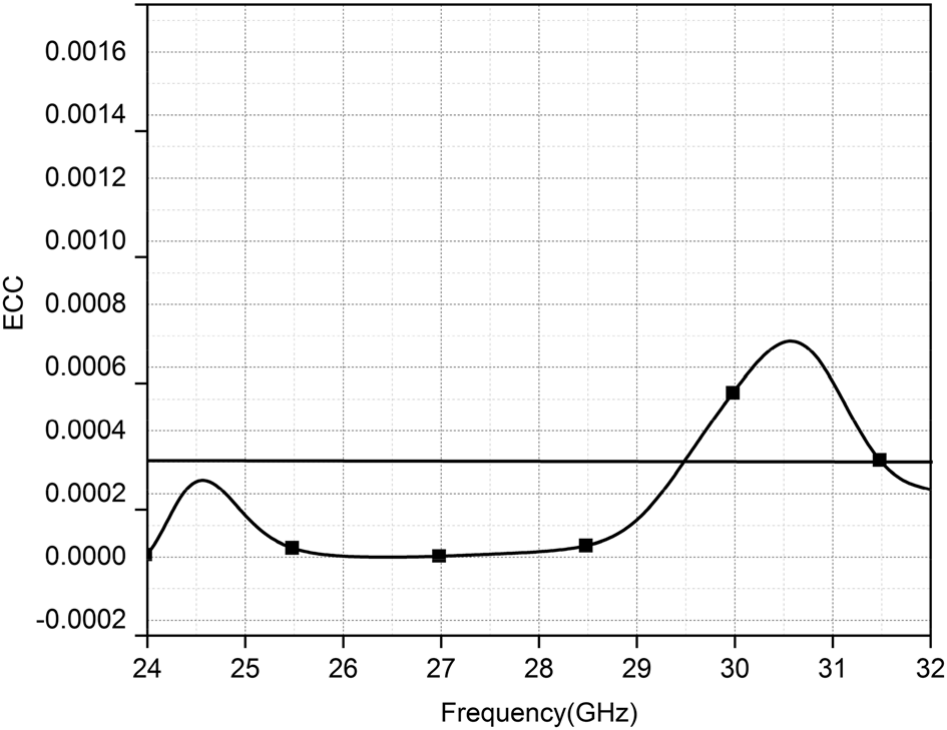
Envelop correlation coefficient of MIMO.

The envelop correlation is a significant parameter as it indicates the antennas' MIMO efficiency. The envelope correlation coefficient determines the independence of MIMO antennas in their individual performance, like polarization diversity, radiation pattern, gain, and phase between the antennas. Figure 14 shows the numerically calculated envelop correlation coefficient for the antennas using^25,26^.

$$ \rho = \frac{{\left| {S_{12} S^{*}_{11} + S_{22} S^{*}_{21} } \right|^{2} }}{{\left[ {1 - \left( {\left| {S_{11} } \right|^{2} + \left| {S_{21} } \right|^{2} } \right)} \right]\left[ {1 - \left( {\left| {S_{22} } \right|^{2} + \left| {S_{21} } \right|^{2} } \right)} \right]}} $$

The maximum value of ρ is lesser than 0.00025 in the entire operating band of the proposed MIMO, which is far less than the practically suitable value referring to the excellent diversity performance of the proposed antenna.

### Diversity gain

The effects of the diversity scheme on radiation power can be illustrated by another fundamental parameter which is known as diversity gain (DG). The following already published relation is used to compute the DG of the proposed MIMO as a function of frequency.

$$DG=10\sqrt{1-|{{\rho }_{eij}|}^{2}}$$

**Figure 15:**
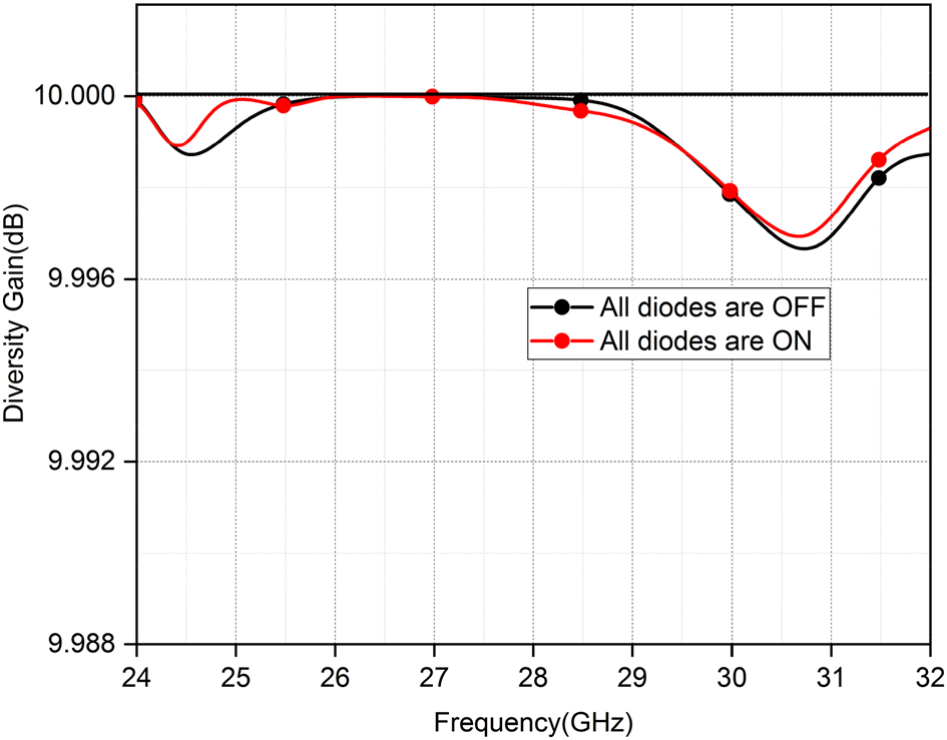
Diversity gain.

Maximum DG for both antenna arrays is 10 dB for the ON and OFF states of the diodes as shown in Fig. 15. The DG slightly drops from 27.9 and 28.5 GHz in the ON and OFF states of diodes, respectively.

**Figure 16:**
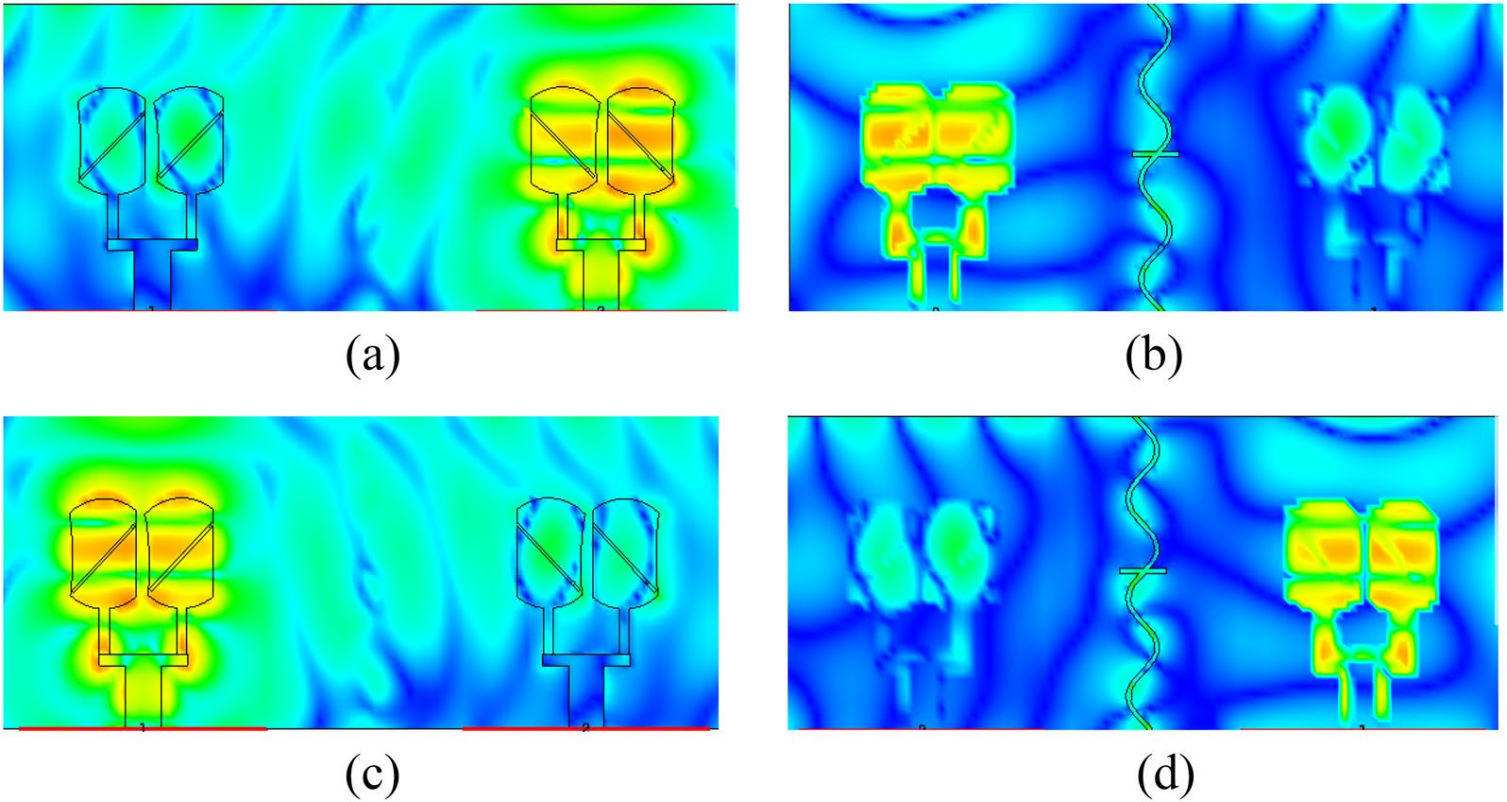
Surface current distribution of proposed MIMO (**a**, **b**) front and back view of array1 (**c**, **d**) front and back view of array2.

Further to investigate the radiation characteristics of the proposed MIMO, the surface current density is presented in Fig. 16a–d. Coupling between array-1 and array-2 is minimum due to slots inserted in the ground. If we observe the current distribution in the back view of the proposed MIMO there is negligible coupling which is due to the insertion of the novel sin wave- like slot in the ground.

### Mean effective gain

In a multipath environment, the statistical measurement of antenna performance is inferred from mean effective gain (MEG). MEG is a ratio of mean received power to the mean incident power at the antenna, and it can be calculated from equation^39^.

$$5{\rm MEGi} = 0.5 \left[[1-{\left|{S}_{ii}\right|}^{2}-{\left|{S}_{ij}\right|}^{2}\right]$$

$${\rm MEGj} = 0.5 \left[[1-{\left|{S}_{ij}\right|}^{2}-{\left|{S}_{jj}\right|}^{2}\right]$$

The practical standard values for MEG ≥ − 3 dB. The calculated values of MEG of proposed MIMO antennas validate this standard as tabulated in Table 2.

**Table 2:** Parameters of proposed design.

Mean effective gain (dB)		
Frequency (GHz)	Antenna#1	Antenna#2
26.59	− 6.9447	− 11.3994
26.98	− 6.73991	− 11.9792
27.49	− 6.8596	− 11.7377
27.97	− 7.0382	− 9.65405
28.57	− 7.34216	− 7.7237
28.99	− 7.58047	− 7.57735
29.08	− 7.63047	− 6.31083
29.5	− 7.8539	− 5.55693

## Measured results

**Figure 17:**
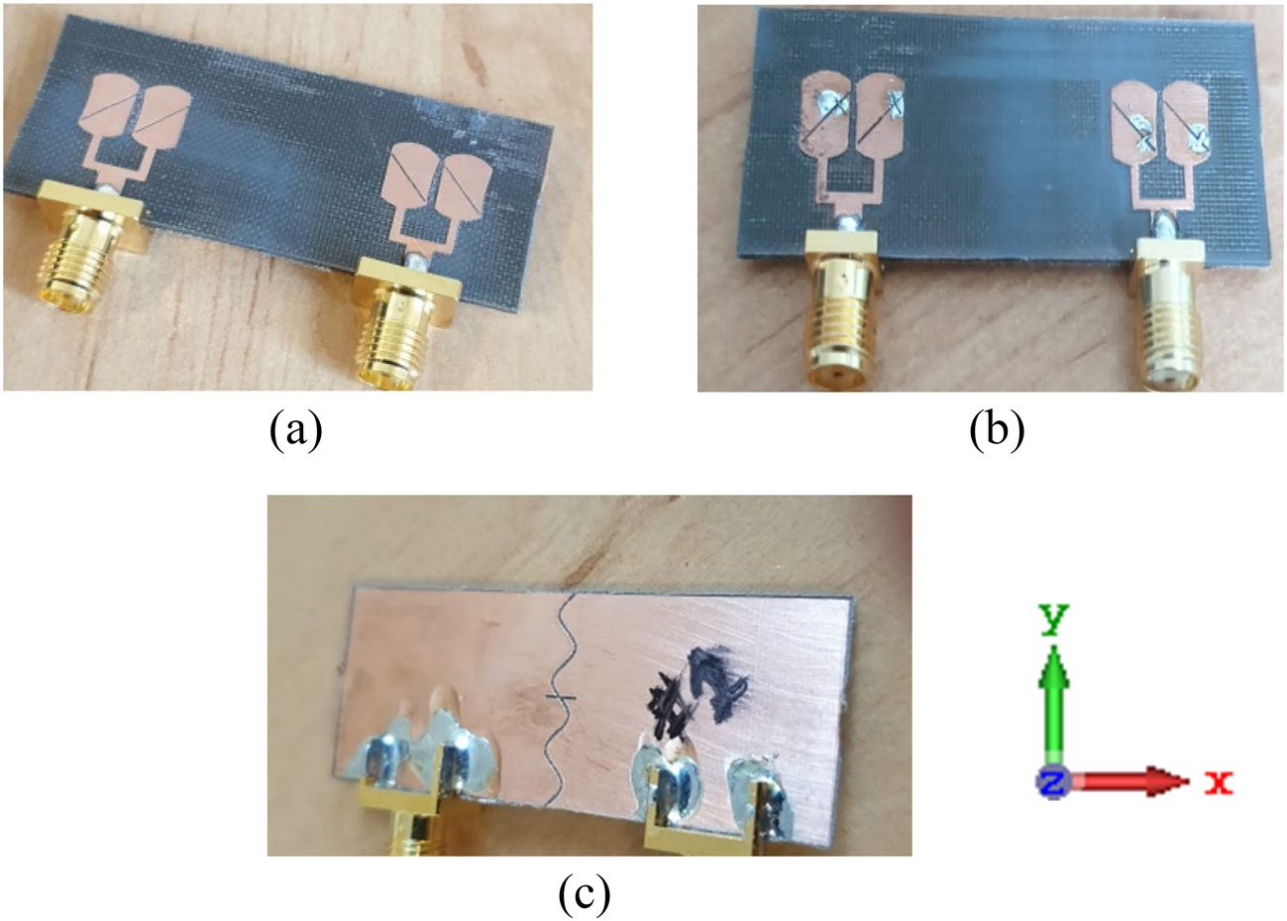
Fabricated antenna (**a**) front view (OFF-state), (**b**) front view (ON-state), (**c**) back view.

The proposed MIMO design is fabricated and measured to validate the performance parameters. Figure 17a–c illustrates the fabricated design, front view in OFF, ON stats of diodes, and back view of the proposed design.

Measurements are taken for four parameters, reflection coefficient, transmission coefficient, AR, and radiation pattern. Prototypes validate the simulated results instead of presenting the final realistic scenario. Real Pin diodes are integrated with the fabricated design as it remains highly challenging to control the accuracy when soldering the switches between such minuscule slots in the array elements. Simply the edges of slots are shorted out.

**Figure 18:**
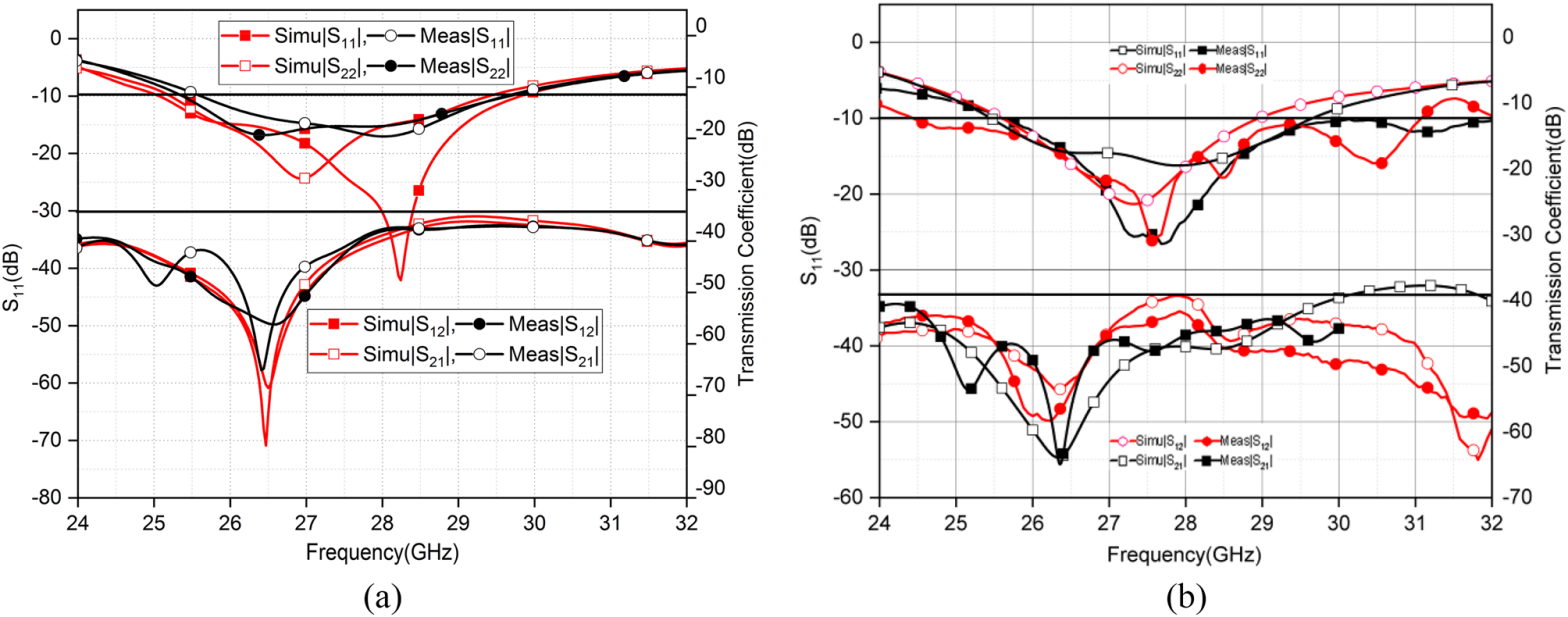
(**a**, **b**) Contrast of simulated and measured reflection and transmission coefficient without and with diodes respectively.

PNA-X Network Analyzer (Eyesight Technologies N5244A) is utilized to measure the S-parameter of the fabricated prototypes. Figure 18a illustrates the simulated and measured S-parameter in the OFF state of all diodes. It is observed that the measured S parameter undergoes a shift. A significant drift is observable in the case of array2. The values, however, remain between the required bands. The expected simulated impedance bandwidth (< − 10 dB) extends from 25.3 to 29.5 GHz. The shared measured impedance bandwidth (< − 10 dB) spans from 25.4 to 29.5 GHz. The Fig. 18b shows the measured and simulated S parameter in the ON state of the diodes. The fabrication limitations posed by a milling machine, and substrate permittivity variations at higher frequencies caused the slight difference between measured and simulated results. The measured results of |S_12_| and| S_21_| have more than 30 dB isolation over the entire operating frequency band and demonstrated strong alignment with simulated results.

**Figure 19:**
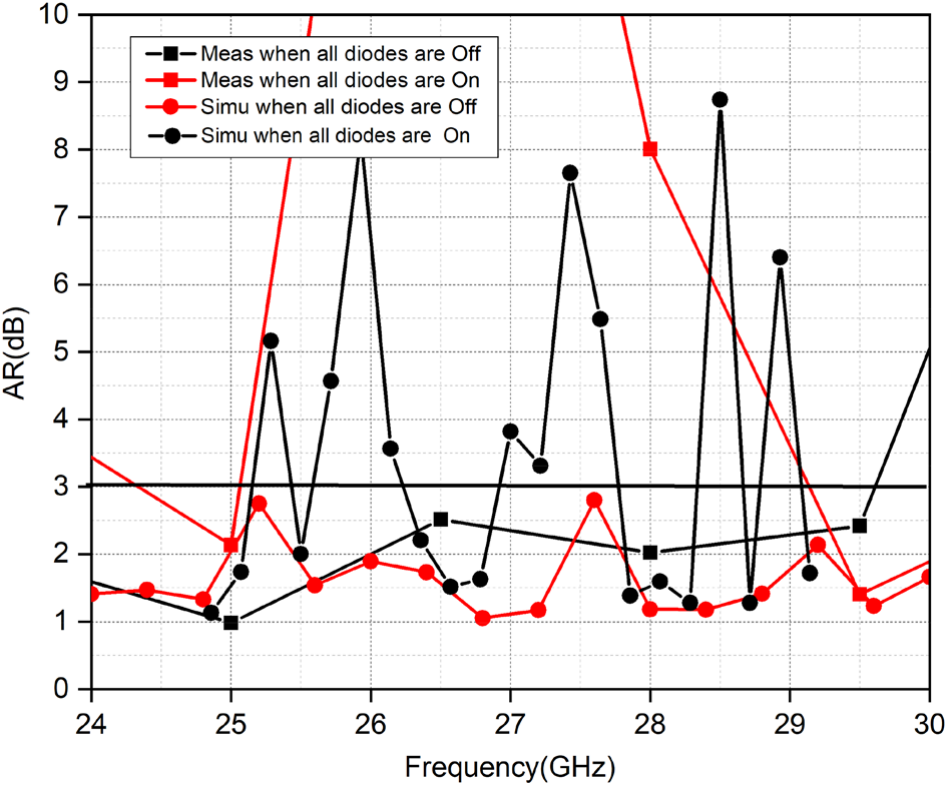
Comparison of measured and simulated AR in both states of Diodes.

Figure 19 shows the measured AR of the proposed MIMO. The simulated and measured AR values in both diode states are in accordance. When all diodes are ON the MIMO elements generate linear polarization. In the OFF state of diodes, the MIMO elements generate CP polarization and cover the whole operating band of 5G.

**Figure 20:**
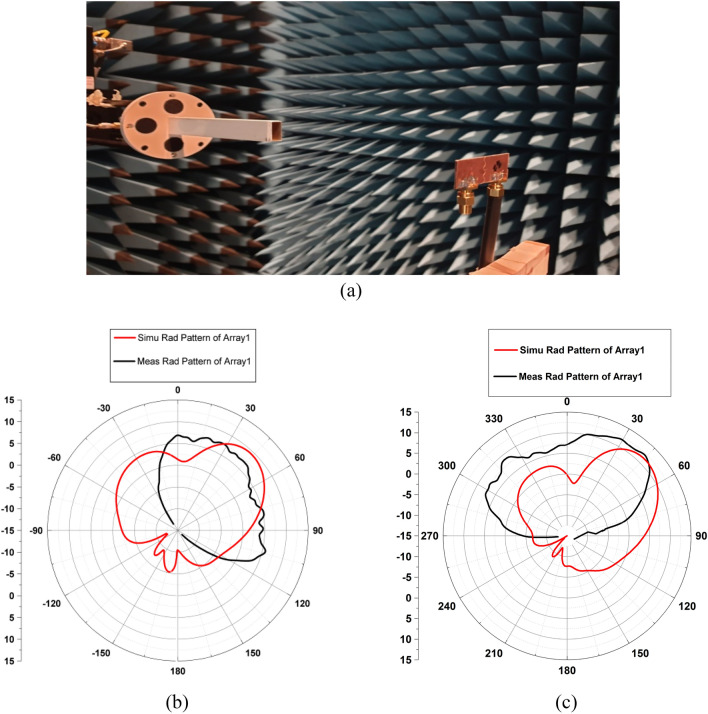
(**a**) Measurement setup (**b**, **c**) XZ plane (H Plane) radiation pattern of MIMO elements in OFF and ON states of diodes.

The radiation pattern is measured using the radiation pattern measurement setup, as shown in Fig. 20a. The Fourier transform algorithm converts the measured radiating energy in the near- field region to far-field^40^. Figure 20 depicts the H- plane simulated and measured radiation patterns at 27.5 GHz for MIMO array elements in the ON and OFF states of the diodes. In particular, the radiation pattern is measured for φ values ranging from 0° to 180° and 0° to -180°. The inconsistencies were observed due to cable losses and fabrication errors. It is also observed that the beam width of the experimentally measured radiation pattern for each antenna array is larger than the simulated results.

Table 3 presents the simulated and measured realized gain of the presented MIMO against the frequencies and MIMO array elements. The gain comparison method is used^41^ for measured gain. The measured characteristics of the MIMO are compared with that of the standard horn antenna to measure the gain of AUT. The maximum gain is 11.3 dBi, which agrees with the simulated gain at 27.5 GHz.

**Table 3:** Simulated and measured gain.

Frequency (GHz)	MIMO Antenna#	Peak gain (dBi)	
		Simulated	Measured
27.5	1	11.4	11.2
27.5	2	11.4	11.2
28	1	10.6	10.5
28	2	10.9	10.7
25.7	1	11.2	11.1
25.7	2	11.2	11.1

Recently different MIMO antenna designs meant for mm-wave are presented in the literature as shown in Table 4. The novelty of the proposed design can be observed from Table 4 that no MIMO design presents the reconfiguration capability. Another notable feature of the proposed design is that its impedance bandwidth and AR bandwidth are equal and the design is quite simple. In^26^ impedance bandwidth and AR bandwidth are similar, but the structure is intricate. Other methods also present CP MIMO configuration at the cost of intricate structure. The proposed design also demonstrates good gain to different designs in the table except^27^, which is designed for Base stations.

**Table 4:** State of the art comparison.

Ref design	Antenna Type	f_c_ (GHz)	Ant Size(mm)	No. of ports	LP/CP	AR BW (%)	|S_11_| BW (%)	Max gain (dBi)	Isolation (dB)	Re- Config
^26^	Fabry- Perot	30	–	4	CP	5	5	8	–	Nil
^27^	Fabry- Perot	28	19 × 19	4	CP	17	27.6	14.1	–	Nil
^28^	Patch	25.2	20.4 × 20.4	8	LP	–	15.6	8.7	30	Nil
^29^	FSS multilayer	30	40.9 × 40.9	4	CP	6	19.3		–	Nil
^30^	Vivaldi	28/38	11.7 × 5.3	12	CP/LP	3	6	8	–	Nil
^42^	Dipole	28	11.3 × 31	4	LP	–	17.5	10	–	Nil
^43^	Patch + EBG	24	48 × 31	2	LP	–	3	6	40	Nil
^44^	Cross dipole	28	–	–	CP	8	8	2.2	–	Nil
^45^	Patch multilayer	28	20 × 8	2	CP	8.3	13.2	11.9	40	Nil
^46^	Air filled slot	28	45 × 45	2	LP	–	0.4	9.7	30	Nil
^47^	Dielectric resonator	28	25 × 15	2	LP	–	0.85	8	24	Nil
^48^	1 × 4 dielectric resonator	30	126 × 126	2	LP	–	1	> 7	25	Nil
^49^	Substrate integrated dielectric Resonator	28	30 × 16	2	LP	–	8.1	9.23	30	Nil
This work	Patch array	28	50 × 12	2	CP/LP	10.7	10.7	11.4	65	Yes

## Conclusion

A polarization reconfigurable MIMO antenna is presented. The design occupied the 26.5 to 29.5 GHz frequency band, which is the n257 band specified by 3GPP in release 15’ for 5G mm-wave communications. Wide impedance bandwidth (3 GHz) and 3 dB AR is achieved in this design. It is worth noting that the whole worldwide 5G mm-Wave frequency band (26.5–29.5 GHz) is cap by the operational CP bandwidth of the PRAA. The two elements of an array are formed, for gain enhancement and maximum gain of 11.4 dBi is achieved. Then the two-port MIMO system is designed and for mutual coupling reduction between the MIMO elements DGS is utilized. So, a kind of new slot, a sin wave, is introduced in the ground. The transmission coefficients show that both ports are well isolated. The measured results of |S_12_| and| S_21_| have more than 30 dB isolation over entire operating frequency band. Other performance metrics of the MIMO system are according to the standard. Polarization diversity is introduced in the MIMO system by incorporating the Pin-Diodes. Design work as a linearly polarized system when diodes are OFF and RHCP when diodes are ON. The design is fabricated to validate the simulated results. The simulated performance parameters of MIMO are in accordance with the experimental results.

## Data Availability

All data generated or analyzed during this study are included in this published article.
